# Seasonal change and influence of environmental variables on host-seeking activity of the biting midge *Culicoides sonorensis* at a southern California dairy, USA

**DOI:** 10.1186/s13071-024-06290-w

**Published:** 2024-05-10

**Authors:** Xinmi Zhang, Jun Li, Alec C. Gerry

**Affiliations:** 1 Department of Entomology, University of California Riverside, Riverside, CA USA; 2 Department of Statistics, University of California Riverside, Riverside, CA USA

**Keywords:** Diel activity, Host-seeking, *Culicoides*, Linear regression, Crepuscular

## Abstract

**Background:**

As a primary vector of bluetongue virus (BTV) in the US, seasonal abundance and diel flight activity of *Culicoides sonorensis* has been documented, but few studies have examined how time of host-seeking activity is impacted by environmental factors. This knowledge is essential for interpreting surveillance data and modeling pathogen transmission risk.

**Methods:**

The diel host-seeking activity of *C. sonorensis* was studied on a California dairy over 3 years using a time-segregated trap baited with CO_2_. The relationship between environmental variables and diel host-seeking activity (start, peak, and duration of activity) of *C. sonorensis* was evaluated using multiple linear regression. Fisher’s exact test and paired-sample z-test were used to evaluate the seasonal difference and parity difference on diel host-seeking activity.

**Results:**

Host-seeking by *C. sonorensis* began and reached an activity peak before sunset at a higher frequency during colder months relative to warmer months. The time that host-seeking activity occurred was associated low and high daily temperature as well as wind speed at sunset. Colder temperatures and a greater diurnal temperature range were associated with an earlier peak in host-seeking. Higher wind speeds at sunset were associated with a delayed peak in host-seeking and a shortened duration of host-seeking. Parous midges reached peak host-seeking activity slightly later than nulliparous midges, possibly because of the need for oviposition by gravid females before returning to host-seeking.

**Conclusions:**

This study demonstrates that during colder months *C. sonorensis* initiates host-seeking and reaches peak host-seeking activity earlier relative to sunset, often even before sunset, compared to warmer months. Therefore, the commonly used UV light-baited traps are ineffective for midge surveillance before sunset. Based on this study, surveillance methods that do not rely on light trapping would provide a more accurate estimate of host-biting risk across seasons. The association of environmental factors to host-seeking shown in this study can be used to improve modeling or prediction of host-seeking activity. This study identified diurnal temperature range as associated with host-seeking activity, suggesting that *Culicoides* may respond to a rapidly decreasing temperature by shifting to an earlier host-seeking time, though this association needs further study.

**Graphical Abstract:**

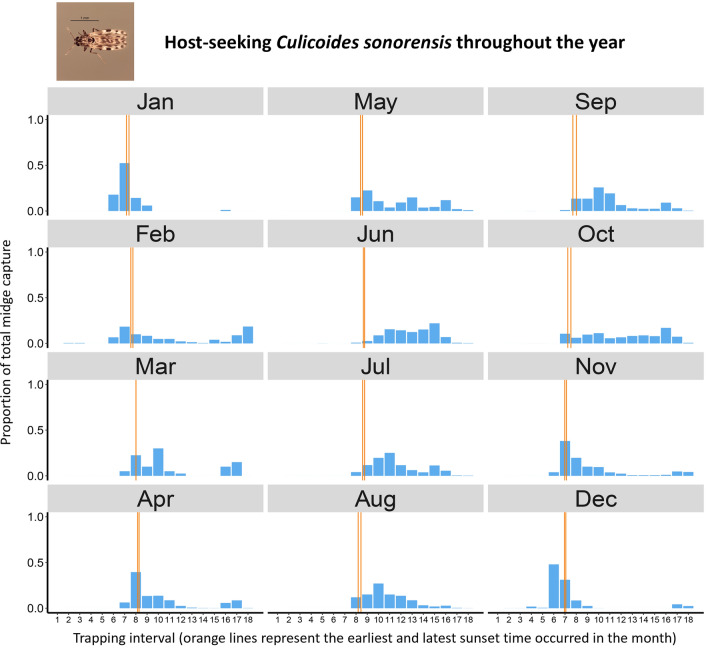

**Supplementary Information:**

The online version contains supplementary material available at 10.1186/s13071-024-06290-w.

## Background

As the primary vector of bluetongue virus (BTV) in North America [1], the geographical and seasonal distribution of *Culicoides sonorensis* Wirth & Jones (Diptera: Ceratopogonidae) has been extensively studied [2–6]. In the western US, *C. sonorensis* is the predominant biting midge species on dairies and is the main vector species responsible for the transmission of BTV to cattle [7]. The activity of *C. sonorensis* is highly seasonal, with midge abundance being substantially greatest in summer and early fall [2, 3] and with BTV infection prevalence in cattle being greatest in fall shortly after peak abundance of *C. sonorensis* [8].

Many *Culicoides* species are generally crepuscular in their host-seeking activity, with peak activity near sunset and/or sunrise and with little or no host-seeking activity during daylight hours [9]. However, flight activity including host-seeking activity has been reported to occur prior to sunset during colder months for some *Culicoides* species [2, 10–12]. By shifting host-seeking activity to before sunset on colder days, midges could seek hosts at a time when environmental conditions were more suitable for flight [13]. Like most *Culicoides* species, peak host-seeking activity of *C. sonorensis* occurs primarily near sunset or occasionally near sunrise in warmer months, with more limited activity throughout the night [2, 10, 13, 14]. In Colorado, Barnard and Jones [2] used truck-mounted traps to show peak flight activity of male and female *C. sonorensis* occurs after sunset in summer but before sunset in spring and fall, suggesting a shift in the timing of midge flight activity associated perhaps with changing temperature or daylength. However, capture rates in this study reflected general flight activity rather than host-seeking flight activity since truck-mounted traps capture flying insects indiscriminately, including those flying for purposes other than host-seeking.

Host-seeking by *C. sonorensis* in southern California also generally begins near sunset during the fall months when midge activity is greatest [10]. However, in northern California host-seeking was reported to occur prior to sunset on a few dates in late winter (February and March) [15], suggesting that host-seeking flight activity by *C. sonorensis* might shift from near sunset during warmer summer and fall months to before sunset during colder winter months.

Environmental and meteorological factors such as temperature, solar radiation, moonlight, and wind speed can affect the flight activity of *C. sonorensis* [2, 3, 14, 16]. Suitable temperature for *C. sonorensis* flight activity is 7–37 ℃ [2], though flight activity may be limited at temperatures < 13 ℃ [13]. Wind speed may be negatively correlated with midge flight activity [16, 17], with flight activity generally suppressed at wind speeds > 2-6 m/s [9, 12, 17]. *Culicoides* host-seeking activity appears to be positively related to moonlight intensity as midges were captured in greater numbers and over a longer period of the night when moonlight was visible [13, 14]. Nevertheless, knowledge of how *C. sonorensis* diel host-seeking activity is influenced by environmental variables is still greatly lacking.

While there has been progress toward understanding *Culicoides* activity as it relates to environmental conditions, there remain substantial knowledge gaps in our understanding of environmental effects on adult host-seeking and how these impacts might alter surveillance outcomes [18]. For example, suction traps baited with UV light are widely used for monitoring *Culicoides* flight activity as part of epidemiological and transmission risk studies, yet light traps have important limitations in this regard [19], including being ineffective during daylight hours. If a shift to pre-sunset host-seeking activity is common on cold days or during cooler winter months, *Culicoides* host-seeking activity may be underreported on these dates when monitoring activity using light traps. Under-reporting of host-seeking activity could lead to underestimation of pathogen transmission risk.

In this study, diel host-seeking activity of *C. sonorensis* in southern California was recorded over a 24-h period every other week for 3 years using a time-segregated trap baited with CO_2_. Trapping data were analyzed to determine whether host-seeking activity occurs earlier during cold winter months as compared to warm summer months. Moreover, we explored how environmental variables influenced host-seeking activity, specifically the start of host-seeking, the time when peak host-seeking occurred, and the duration of the host-seeking period.

## Methods

### Study site

**Fig. 1:**
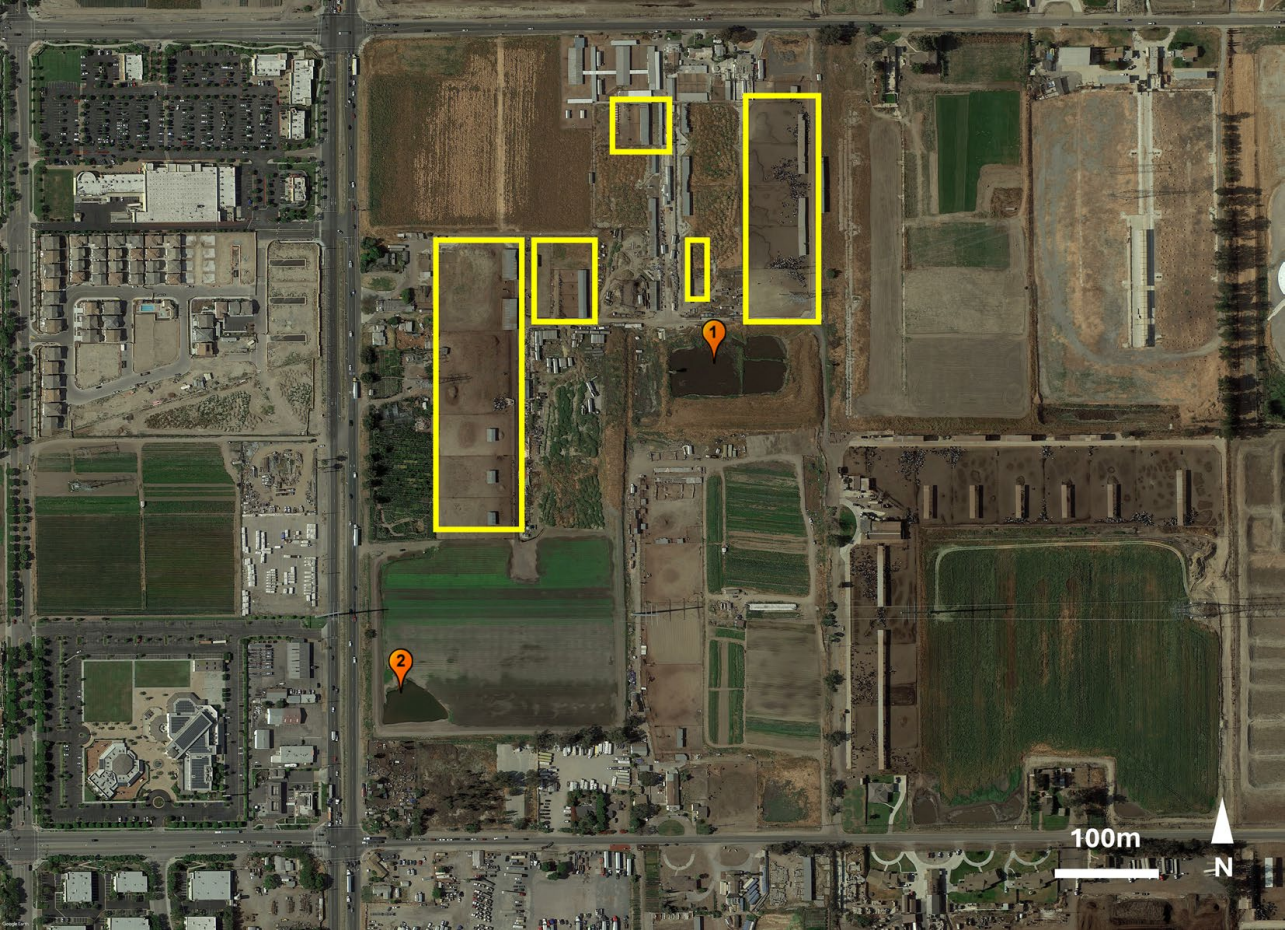
Google earth image showing the study site. Yellow rectangles indicate cattle pens at the study dairy. Wastewater ponds that were development sites for immature *Culicoides sonorensis* are indicated by pin 1 (north pond) and 2 (south pond). The north pond was larger than the south pond at the time of study. There were several other dairies within 2 km to the north and 8.5 km to the east of this dairy, which sits at the western edge of the former Chino Dairy Preserve in southern California

This study was conducted over 3 years (2018–2021) at a drylot dairy in the Chino dairy region of southern California (San Bernadino County, CA, USA). The study dairy maintains a herd of > 1000 cows in multiple cattle pens with two retention ponds to capture and retain wastewater from milking operations (Fig. 1). These manure-polluted wastewater ponds are a common immature development site for *C. sonorensis* [3, 20]. The larger northernmost pond (north pond) was heavily vegetated during year one of the study, but was partially drained, dredged, and cleared of some of the vegetation in year 2 to reduce mosquito development per requirement of the local health authority. The smaller southernmost pond (south pond) was shallow with gently sloping sides and lacked vegetation. A high density of immature *Culicoides* was commonly observed along the edges of the south pond during late summer and early fall of each study year. During fall of year 3, the flow of wastewater to the south pond was stopped resulting in loss of this pond due to evaporation by late fall. Two smaller farms housing a small number of various animal species including horses, sheep, goats, pigs, and fowl were adjacent to the study dairy to the south and southeast.

#### Insect collection

Host-seeking midges were captured for one 24-h collection period every other week for 3 years (April 2018–April 2021). During each trap week, the collection date was selected to avoid predicted precipitation, but environmental conditions were otherwise not considered in timing of the collection date. Midges were captured using a single battery-operated, time-segregated rotating trap with 18 collection jars containing ~ 50 ml of soapy water to retain captured insects and which rotated in sequence beneath a Centers for Disease Control (CDC) type miniature suction trap without light (Model 512, J.W. Hock, Gainesville, FL) [10]. Carbon dioxide (CO_2_) from a compressed gas tank was released near the trap opening at a flow rate of 1000 ml/min to mimic the breath of a nearly grown Holstein heifer [8, 21]. The time-segregated trap was set on a small table positioning the trap opening ~ 1.3 m above ground.

Each of the 18 collection jars rotated into position beneath the trap for an 80-min trapping interval to complete the 24-h collection period. Trapping began at 8:45 a.m. (9:45 a.m. during Daylight Saving Time [DST]) on each collection date. To normalize trapping intervals relative to sunset across collection dates, the trapping interval that included sunset was coded as interval 0 with trapping intervals before sunset coded as sequentially decreasing negative numbers (− 1, − 2, − 3, …) and trapping intervals occurring after sunset coded as sequentially increasing positive numbers (1, 2, 3, …).

The time-segregated trap was positioned near the north pond from April 2018 through March 2019, shifted to near the south pond from April 2019 through October 2020 because of physical alteration of the north pond during the 2nd year of the study and then returned to near the north pond through the end of the study. Insects captured in each collection jar were sorted, and *C. sonorensis* were counted by sex and physiological status of females (nulliparous, parous, blood-engorged, or gravid) [22, 23].

#### Environmental and weather information

Weather information including temperature, relative humidity, wind speed, and solar radiation was acquired from a nearby weather station ca. 2.5 km away (Station KCACHINO13: 34.01°N, 117.67°W) with data accessed using WeatherUnderground [24]. Weather data from this weather station were unavailable on three collection dates during the study period, so the weather information on these dates was obtained instead from two other nearby weather stations (Station KCACHINO71: 34.02°N, 117.69°W, ca. 5 km away or Station KCACHINO89: 34.01°N, 117.67°W, ca. 2.5 km away). All weather stations collected data every 5 min. Time of sunset and sunrise, as well as the moon phase for each collection date, was obtained from Timeanddate.com [25].

#### Data analysis

Analyses and visualization of the collection data were conducted in R version 4.2.2 [26]. Since CO_2_-baited traps were used for midge collection, empty midges regardless of parity were all considered host-seeking. Host-seeking activity during each 24-h collection period was analyzed as (1) start of host-seeking, (2) time of peak host-seeking activity, and (3) host-seeking duration. Start of host-seeking is the first trapping interval after midday during which midges were captured followed by increasing captures in subsequent intervals. Time of peak host-seeking activity is the trapping interval during which the greatest number of midges were captured between start of host-seeking and sunrise the next morning. If the peak number of host-seeking midges was the same during two contiguous trapping intervals (occurred on three dates over the 3-year study) then the time of peak activity was recorded as the numerical mean of the two trapping interval codes. Host-seeking duration is the number of continuous trapping intervals in which host-seeking midges were captured after the start of host-seeking activity through sunrise the following morning.

Regression analyses were used to study the relationship between environmental variables and host-seeking activity. Independent variables considered in the analyses were the high (maximum) and low (minimum) temperature during the 24 h prior to start of the collection period (T_h-1_, T_l-1_) and during the collection period (T_h_, T_l_), high and low relative humidity during the 24 h prior to start of the collection period (R_h-1_, R_l-1_) and during the collection period (R_h_, R_l_), high solar intensity during the collection period (S_h_), high wind speed during the collection period (W_h_), and the temperature, relative humidity, and wind speed at sunset on each collection date (T_s_, R_s_, W_s_) as well as moon phase represented by the number of days to the closest new moon (M_n_). Temperature and relative humidity during the 24 h prior to the start of the collection period were considered in the analysis as these variables were thought to have potentially impacted the number of midges available to host-seek during the collection period. Since collections were conducted on days without rain, precipitation was not considered in the analyses.

We selected potentially important environmental variables using three variable selection methods provided by R package “olsrr” [27]: forward, backward, and stepwise selection. Since different variable selection methods may select different environmental variables, for each variable selection method, we applied the following model-building procedure: (1) build the model with only first-order terms selected by the variable selection method and remove terms with high variance inflation factor (vif > 5) to avoid multicollinearity; (2) build a complete second-order model using the first-order terms that remained after step 1 and adding quadratic terms and interaction terms; (3) remove non-significant terms in the complete second-order model step by step based on their *P*-value until all terms remaining in the model had *P* < 0.05; (4) perform residual analysis to confirm model assumptions are satisfied. In addition, we built a model following the same procedure as above but using all variables identified as significant by any of the three selection methods. Models with good residual analysis were then compared to find the best fit model based on the least Akaike information criterion (AIC) and evaluated by leave-one-out cross-validation.

Start of host-seeking and time of peak host-seeking activity were further evaluated using Fisher’s exact test for a shift in the frequency that these activities occurred before, during, or after sunset in cooler months (November–April) as compared to warmer months (May–October) combining data across all 3 years. Differences in host-seeking activity by midge parity (nulliparous vs. parous) were determined by a paired-sample z-test for start of host-seeking, time of peak host-seeking activity, and host-seeking duration.

## Results

### Host-seeking activity relative to sunset

**Fig. 2:**
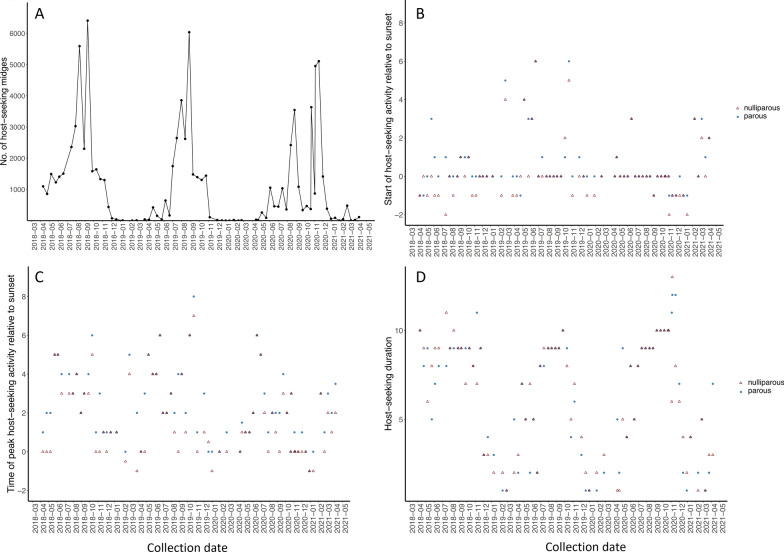
Collection of host-seeking *Culicoides sonorensis* throughout the 3-year study. (**A**) Number of host-seeking midges captured; (**B**) start of host-seeking, (**C**) time of peak host-seeking activity; (**D**) duration of host-seeking activity. In panels **B** and **C**, sunset occurred during trapping interval *y* = 0, with a negative number on the *y*-axis indicating a collection period before sunset. In panel **D**, duration is the number of consecutive 80-min collection intervals with host-seeking activity on each date

**Fig. 3:**
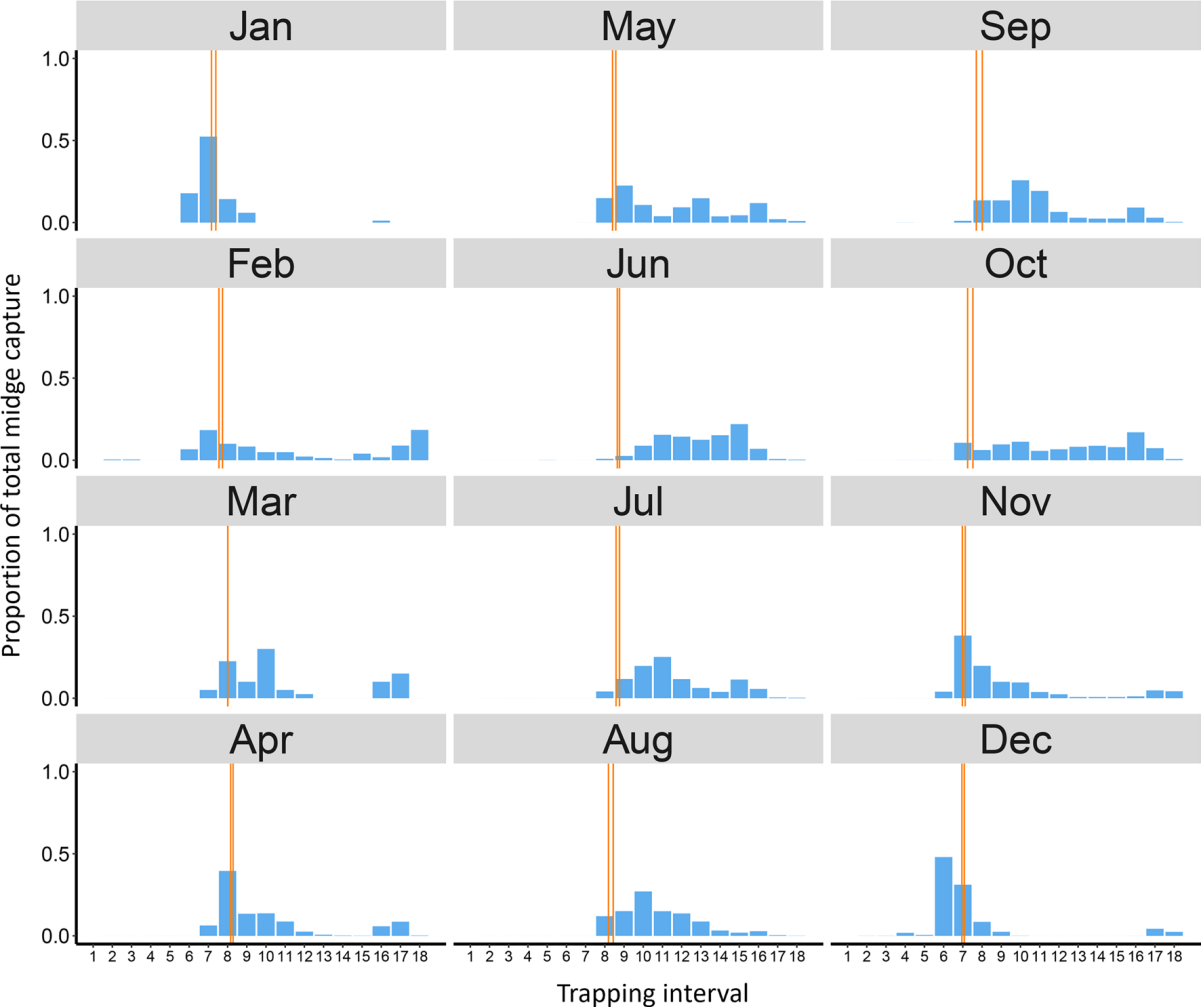
A summary of host-seeking *Culicoides sonorensis* throughout the year. Columns show host-seeking midges (parous and nulliparous combined) captured during each 80-min trapping interval (1–18) as a mean of the proportion of the total number of midges captured on each collection date during the month. Trapping began at 8:45 a.m. on each collection date. Two orange vertical lines represent the earliest and latest sunset time that occurred during the month

A total of 98,229 *C. sonorensis* were captured over 77 collection dates from April 2018 through April 2021, comprising 43,963 parous females (45% of all *C. sonorensis* collected), 43,124 nulliparous females (44%), 1028 blood-fed females (1%), 13 gravid females (< 0.01%), and 10,101 males (10%). Parous and nulliparous females captured in the trap were considered host-seeking since the trap was baited with CO_2_ and did not have a light source. The number of host-seeking midges captured changed drastically throughout the year with peak activity during late summer through early fall and with low activity from December through April (Fig. 2A). The greater capture of host-seeking midges recorded in November 2020 relative to the previous month coincided with a change in the trap location at the dairy from near the south pond to near the north pond and thus likely does not represent a true increase in host-seeking activity in the region but rather greater midge abundance near the north pond than near the south pond at that time. Movement of the trapping site has little impact on this study since proportional capture (not the actual number of midges captured) during each trapping interval was used in the analyses of environmental associations relative to the timing of host-seeking (start, peak, duration). During warmer months from late spring through fall (late May to early November), host-seeking activity occurred continuously from near sunset through sunrise with few exceptions, while during cooler winter and early spring months (December through April) host-seeking activity was mostly discontinuous across nighttime trapping intervals with few or no midges captured during many of the late night or very early morning trapping intervals (Fig. 3; Additional file 1: Figs. S1-S3). The few gravid females were captured between May–November from one to five intervals after sunset (11 individuals) or one to three intervals after sunrise (2 individuals).

### Environmental predictors associated with diel host-seeking activity

#### Time of peak host-seeking activity

**Fig. 4:**
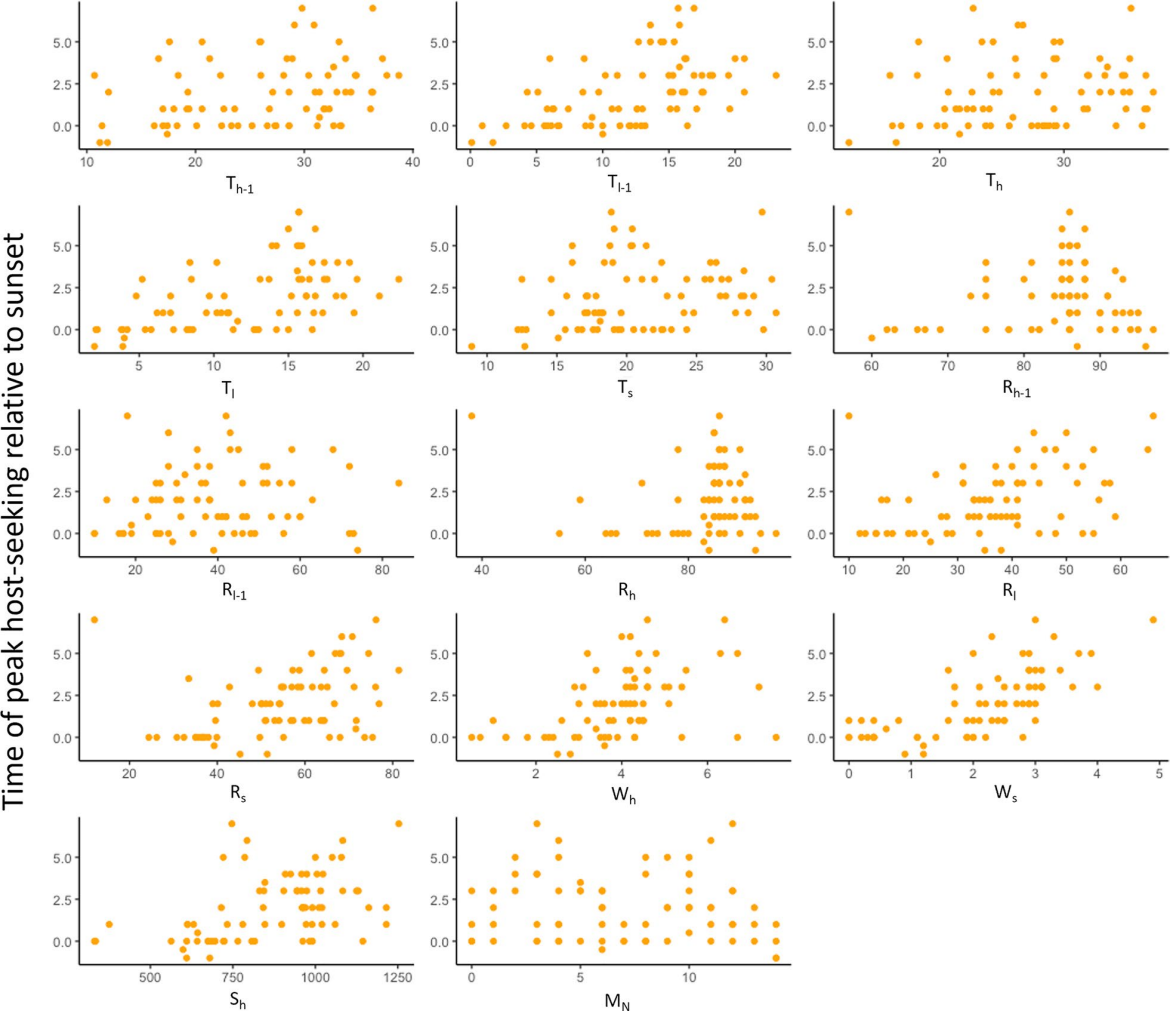
Relationship between environmental variables and time of peak host-seeking activity relative to sunset (*y* = 0). Independent variables considered in the analyses were the high (maximum) and low (minimum) temperature during the 24 h prior to start of the collection period (T_h-1_, T_l-1_) and during the collection period (T_h_, T_l_), high and low relative humidity during the 24 h prior to start of the collection period (R_h-1_, R_l-1_) and during the collection period (R_h_, R_l_), high solar intensity during the collection period (S_h_), high wind speed during the collection period (W_h_), and the temperature, relative humidity, and wind speed at sunset on each trapping date (T_s_, R_s_, W_s_) as well as moon phase represented by the number of days to the closest new moon (M_n_)

**Fig. 5:**
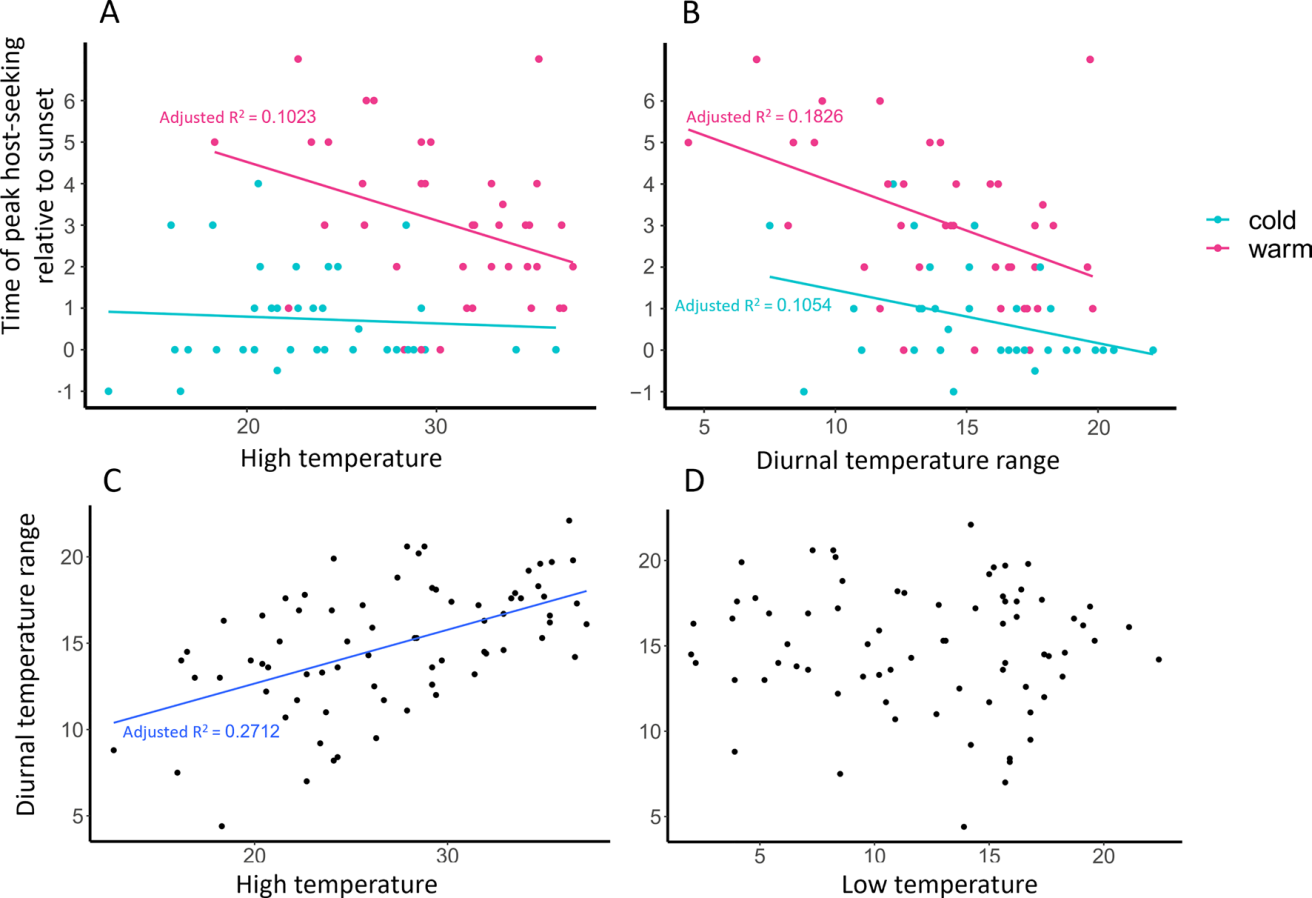
Relationship between temperature and time of peak host-seeking activity. The upper dot plots show the relationship between the time of peak host-seeking activity relative to sunset (*y* = 0) on each collection date and (**A**) the high temperature or (**B**) the diurnal temperature range (difference between the high and low temperature). Dot colors indicate a seasonal association with cold months (blue) being November through April and warm months (red) being May through October. The lower dot plots show the relationship between the diurnal temperature range and (**C**) high temperature or (**D**) low temperature. Adjusted R^2^ values are indicated for regression lines with significantly associated factors

The best model describing time of peak host-seeking activity was obtained by backward selection (adjusted R-squared = 0.6577; *F* = 36.07; *df* = 4, 69; *P* < 0.0001) and included high and low temperature during the collection period and wind speed at sunset (Table 1, Fig. 4). The variables and their range in the analysis are provided in Additional file 1: Table S1. The time of peak host-seeking activity was positively associated with low temperature, indicating peak host-seeking activity occurs later on warmer nights. Paradoxically, the time of peak host-seeking activity was negatively associated with high temperature, indicating peak host-seeking activity occurs earlier on hotter days. The negative association between the daily high temperature and time of peak host-seeking activity is particularly evident during warm months (May–October) (*P* = 0.0267) (Fig. 5A). The paradox is perhaps explained by time of peak host-seeking activity being negatively associated with the diurnal temperature range (difference between daily high and low temperature) (*P* < 0.0001) during both warm and cold months (*P* = 0.0039 and 0.0321, respectively) (Fig. 5B). The diurnal temperature range was associated with the high temperature (Fig. 5C) but not the low temperature (Fig. 5D). There is a quadratic relationship between time of peak host-seeking activity and wind speed at sunset with wind speeds below ca. 0.69 m/s associated with peak activity occurring at or near sunset, while greater wind speeds at sunset are associated with peak host-seeking activity being delayed until after sunset.

**Table 1:** Regression coefficients for the best linear regression model describing time of peak host-seeking activity

	Coefficient	Std. error	t value	*P* ( >|t|)	Partial R-square
T_l_	0.29	0.053	5.401	< 0.001	0.30
T_h_	– 0.21	0.04	– 5.36	< 0.001	0.29
W_s_	– 0.48	0.41	– 1.16	0.25	0.02
W_s_^2^	0.35	0.09	3.761	< 0.001	0.17

Model: ŷ = 3.10 + 0.29T_l_–0.21T_h_–0.48W_s_ + 0.35W_s_^2^

*Std. Error* standard error

T_l_: low (minimum) temperature during the collection period; T_h_: high (maximum) temperature during the collection period; W_s_: wind speed at sunset of the collection date

#### Host-seeking duration

**Fig. 6:**
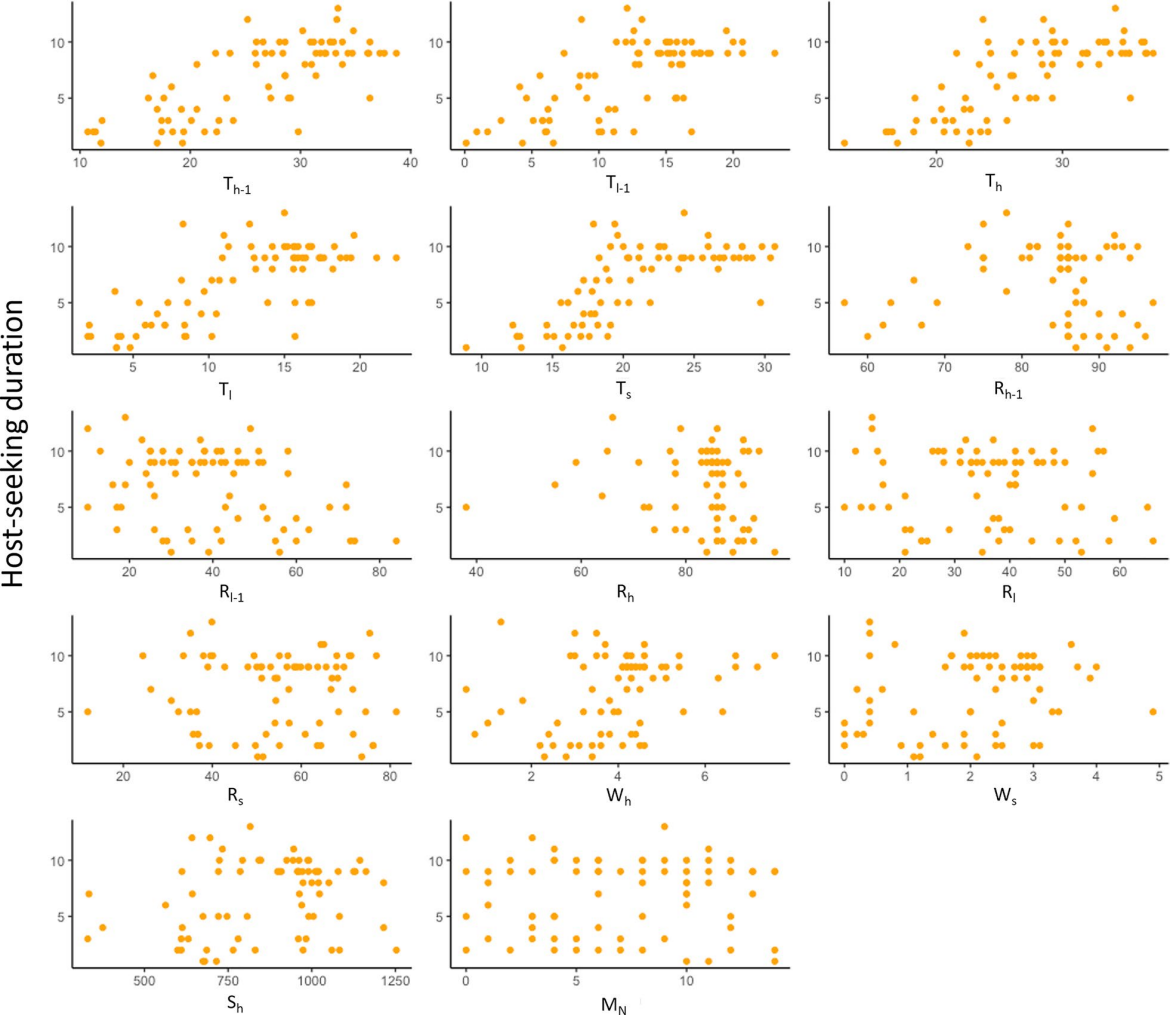
Relationship between environmental variables and host-seeking duration (number of consecutive trapping intervals with host-seeking activity). Independent variables considered in the analyses were the high (maximum) and low (minimum) temperature during the 24 h prior to start of the collection period (T_h-1_, T_l-1_) and during the collection period (T_h_, T_l_), high and low relative humidity during the 24 h prior to start of the collection period (R_h-1_, R_l-1_) and during the collection period (R_h_, R_l_), high solar intensity during the collection period (S_h_), high wind speed during the collection period (W_h_), and the temperature, relative humidity, and wind speed at sunset on each trapping date (T_s_, R_s_, W_s_) as well as moon phase represented by the number of days to the closest new moon (M_n_)

The best model describing host-seeking duration of *C. sonorensis* was obtained using the stepwise selection method (adjusted R-squared = 0.6735; *F* = 38.64; *df* = 4, 69; *P* < 0.0001) (Table 2, Fig. 6). In this model, significant factors were the high and low temperature during the collection period and wind speed at sunset. Host-seeking duration was positively associated with high temperature during the collection period, indicating host-seeking occurs over more continuous nighttime hours on warmer days relative to cooler days. Host-seeking duration had a quadratic relationship with low temperature with host-seeking duration positively associated with low temperature up to ca. 16 ℃, above which further increases in low temperature were not associated with a change in host-seeking duration. Host-seeking duration was negatively associated with wind speed at sunset, suggesting that when higher wind conditions occur at sunset, host-seeking activity is delayed and therefore reduces the overall nighttime period available for host-seeking.

**Table 2:** Regression coefficients for the best linear regression model describing host-seeking duration

	Coefficient	Std. error	*t* value	*P* ( >|t|)	Partial R-square
T_h_	0.22	0.06	3.566	< 0.001	0.16
T_l_	0.97	0.21	4.599	< 0.001	0.23
W_s_	– 0.94	0.26	− 3.611	< 0.001	0.16
T_l_^2^	– 0.03	0.01	− 3.089	< 0.01	0.12

Model: ŷ = – 4.46 + 0.22T_h_ + 0.97T_l_–0.03T_l_^2^–0.94W_s_

*Std. error* standard error

T_l_: low (minimum) temperature during the collection period; T_h_: high (maximum) temperature during the collection period; W_s_: wind speed at sunset of the collection date

#### Start of host-seeking activity

**Fig. 7:**
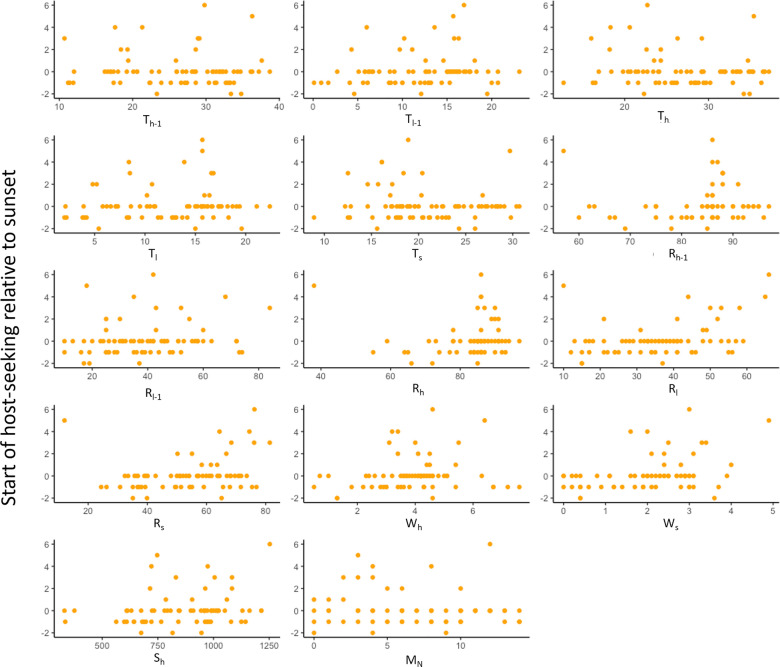
Relationship between environmental variables and start of host-seeking activity relative to sunset (*y* = 0). Independent variables considered in the analyses were the high (maximum) and low (minimum) temperature during the 24 h prior to start of the collection period (T_h-1_, T_l-1_) and during the collection period (T_h_, T_l_), high and low relative humidity during the 24 h prior to start of the collection period (R_h-1_, R_l-1_) and during the collection period (R_h_, R_l_), high solar intensity during the collection period (S_h_), high wind speed during the collection period (W_h_), and the temperature, relative humidity, and wind speed at sunset on each trapping date (T_s_, R_s_, W_s_) as well as moon phase represented by the number of days to the closest new moon (M_n_)

All three selection methods led to the same model for start of host-seeking activity, but this model had a low adjusted R-squared value (adjusted R-squared = 0.4308; *F* = 12.05; *df* = 5, 68; *P* < 0.0001); thus, the model was deemed insufficient to explain timing for the start of host-seeking (Fig. 7). The start of host-seeking ranged from 160 min before to 480 min after sunset over the 3-year study.

#### Seasonal and parity differences in host-seeking activity

**Fig. 8:**
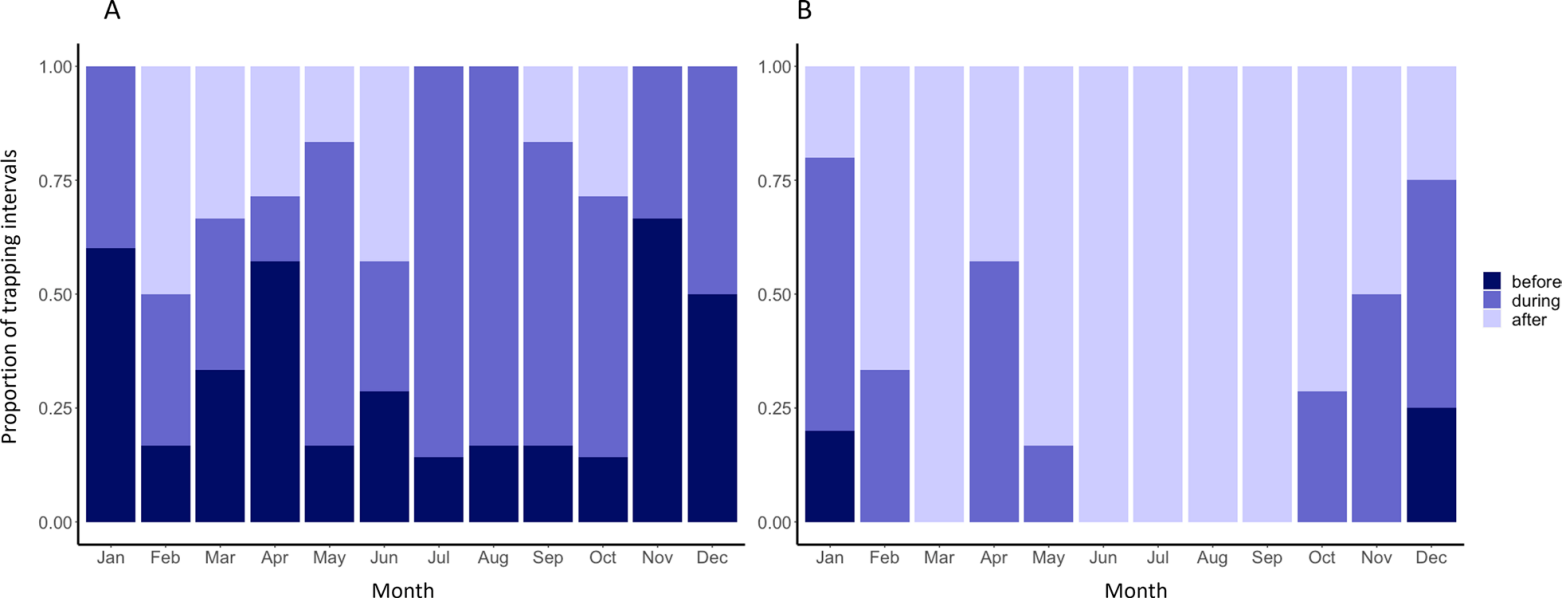
Proportion of trapping intervals during each month (2018–2021) when (**A**) start of host-seeking or (**B**) peak host-seeking activity occurred before (dark blue), during (blue), or after (light blue) the trapping interval that included sunset. Host-seeking activity began before sunset more often during colder months (Nov-Apr). Peak host-seeking activity occurred before or during sunset more often in colder months (November–April)

The frequency with which host-seeking activity occurred before or after sunset varied by season (*P* = 0.006, OR = 0.237, 95% CI 0.068–0.738). Host-seeking activity began slightly earlier during colder months (November–April) relative to warmer months (May–October), often starting before sunset during winter (November–January) while only occasionally starting before sunset from late spring through early fall (May–October) (Fig. 8A). The time when peak host-seeking activity occurred shifted with season even more distinctly, with peak activity occurring before sunset only in December and January while always occurring after sunset during the summer months (June–September) (*P* < 0.0001, OR = 0.073, 95% CI 0.012–0.298) (Fig. 8B).

The time that host-seeking activity occurred differed with midge parity. Parous *C. sonorensis* started host-seeking (z = − 1.99, *P* = 0.02) and reached peak host-seeking activity (z = − 2.47, *P* < 0.01) during a later trapping interval compared to nulliparous midges. Relative to nulliparous midges, parous midges started host-seeking later on 35% of collection dates (Fig. 2B) and reached peak activity later on 49% of collection dates (Fig. 2C). On dates when the activity of parous midges occurred later, the time difference among nulliparous and parous midges was most often just one trapping interval for both start of host-seeking (84% of dates) and time of peak host-seeking (74% of dates). Over all collection dates when parous midge activity was delayed, the delay was 1–3 trapping intervals for the start of host-seeking and 1–4 trapping intervals for the peak activity time. Notably, a later start and peak host-seeking activity for parous midges was observed during both warm and cold seasons. In contrast, the duration of host-seeking activity was not different for parous and nulliparous midges (z = − 0.33, *P* = 0.74) (Fig. 2D).

## Discussion

The seasonal abundance of host-seeking *C. sonorensis* during this study is consistent with previous studies in the same region [3] with seasonal activity being highest in late summer and lowest during winter. The relatively high host-seeking activity (480 midges captured) recorded on one warm wintertime day (February 23, 2021) is not unusual in this region, as host-seeking activity occurs intermittently throughout winter in southern California and may serve as an overwintering mechanism for bluetongue virus which is endemic in the region [28].

There was a distinct seasonal shift in the time of host-seeking activity relative to sunset. Host-seeking activity occurred before sunset more often during colder winter months relative to warmer summer and fall months when host-seeking activity followed the more typical crepuscular pattern previously reported for *C. sonorensis* in southern California [10]. This shift in host-seeking activity is most apparent as an earlier peak in activity during colder winter months, with peak host-seeking occurring before sunset only during December and January while during summer months peak host-seeking always occurred after sunset. Though the start of host-seeking also began at an earlier time relative to sunset during the winter months, this seasonal shift was less distinct, perhaps because of *C. sonorensis* being otherwise constrained by its generally crepuscular habit from initiating host-seeking too far before sunset.

These findings suggest that midges alter their typically crepuscular host-seeking behavior in response to cooling temperature, reduced daylength or other environmental conditions associated with seasonal change, perhaps to ensure that host-seeking activity occurs before low nighttime temperatures restrict flight. In northeastern Colorado, Barnard and Jones [2] observed a similar pattern in overall flight activity (including non-host-seeking flight) for *C. sonorensis*, with flight activity occurring near sunset in July and August while more frequently occurring before sunset during the cooler spring and fall months.

Midge parity impacted both the time host-seeking began and the time peak host-seeking activity occurred, though a delay in host-seeking activity by parous midges occurred on < 50% of collection dates, and on these dates the delay was usually brief (often just one 80-min trapping interval). Environmental factors do not appear to be associated with delayed host-seeking activity by parous midges since differences in midge activity according to parity were observed during both warm and cold months. The later host-seeking activity by parous midges is perhaps a result of oviposition activities by gravid females that occur near sunset before these females can return to host-seeking behaviors [29].

This study expands on previous work showing temperature to be an important factor influencing the flight activity of *C. sonorensis* [2, 12, 14, 16]. Given the seasonal shift in host-seeking activity demonstrated in the current study, it is perhaps not surprising that both low and high daily temperatures are predicators for both the time of peak host-seeking activity and the duration of host-seeking activity.

As low daily temperature decreases, a shift to both an earlier activity peak and a shorter duration of host-seeking would ensure that midges avoid nighttime temperatures that are too low for flight. Temperatures < 7 ℃ are known to restrict *Culicoides* flight [2]. It is notable that during the current study on dates when the low temperature was ≤ 5 ℃, peak host-seeking activity occurred before sunset and thus at a time when temperature was still above the 7 ℃ flight threshold. When low temperatures were ≥ 16 ℃, there was no effect of low temperature on the duration of host-seeking, suggesting midge host-seeking flight is not restricted by temperatures above this.

Increasing high temperature was associated with a longer duration of host-seeking activity as might be expected since nighttime temperatures would not be expected to exceed the high temperature limit for flight activity (37 ℃) reported by Barnard and Jones [2] and because midge abundance in southern California is greatest during the hot summer and early fall months [8]. In contrast, the negative association between high temperature and time of peak activity in the current study seems counterintuitive. However, a higher daytime temperature would be expected to be associated with a more rapid temperature decline during the afternoon and early evening (positively related to diurnal temperature range). A more rapid decline in temperature throughout the afternoon and early evening might signal a potentially very low overnight temperature, thus encouraging earlier host-seeking by midges to avoid a later nighttime temperature that might negatively impact host-seeking. However, the association of diurnal temperature range with host-seeking activity is indirect and should be further investigated to confirm this relationship. The association of daily high temperature with time of peak activity was especially evident during warmer months, with increasing daily high temperature associated with a shift in peak activity from the typical 3–9 h after sunset during these warm months to peak activity occurring closer to sunset. The diurnal temperature range may also have other impacts on midge behavior or physiology that were not examined in the current study. For example, *Aedes aegypti* mosquitoes infected with dengue virus had lower survival and altered vector competence when exposed to a higher diurnal temperature range even when the mean temperature experienced was unchanged [30].

The negative effect of high wind speeds on the flight activity of midge species including *C. sonorensis* has been previously reported [16, 31] with high wind speeds generally associated with a decrease in the biting rate of midges [17]*.* In southern California, wind often changes in a predictable pattern, increasing in mid-late afternoon through sunset, and then rapidly decreasing and remaining low through sunrise the next morning. In the current study, wind speed at sunset was a predictor for both the time of peak host-seeking activity and the duration of host-seeking activity, with wind speeds > 0.69 m/s at sunset associated with a delay in peak host-seeking until winds had abated sufficiently. Wind speeds > 2 m/s at sunset were often associated with peak host-seeking activity occurring one or more trapping intervals after sunset. It might also be expected that high winds near sunset would delay the start of host-seeking, though this could not be confirmed by modeling in this study, resulting in a reduced duration of host-seeking activity as the length of the remaining nighttime period is shortened. While the effect of wind in this study occurs at a lower wind speed than the 2–6 m/s wind speed reported to restrict midge flight [9, 32], it is not unreasonable to consider that midge flight and host location may be impacted at lower wind speeds than those reported to prevent flight.

The time host-seeking activity began each day was not predicted by environmental variables, perhaps because of the relative importance of sunset in initiating host-seeking behavior in these crepuscular insects. During the 3-year study, host-seeking began either very near sunset or during the trapping interval prior to sunset on 58 of 74 (78%) collection dates. As a result, the start time for host-seeking activity did not provide enough variation to show an effect of environmental variables.

Variation in host-seeking activity across seasons and according to environmental variables was most clearly seen as a shift in the time of peak host-seeking activity, perhaps because the time when peak activity occurs is less impacted by outlier midge activity relative to the start time or duration of host-seeking, each of which can be altered by a small number of midges seeking hosts outside the typical time pattern on any given date. Shifts in the time of host-seeking are likely a behavioral response to ensure host-seeking occurs under conditions suitable for flight and orientation toward hosts. During winter, low nighttime temperatures encourage earlier host-seeking activity, including host-seeking prior to sunset when temperature conditions may be more suitable for host-seeking flight. During summer, higher daily temperature and the related increase in the diurnal temperature range also encourage earlier host-seeking, but peak host-seeking in this case still largely occurs after sunset.

## Conclusions

This study showed that *C. sonorensis* host-seeking activity varies by season and can be predicted to some extent by temperature and wind speed. Understanding these impacts on midge host-seeking activity may help to develop more effective methods for midge surveillance and control, including providing a basis for timing protective application of insecticides or repellents to animals. The finding that peak host-seeking activity can shift to occur before sunset in winter can have important implications for midge surveillance. Since *Culicoides* surveillance often relies on the use of UV light traps, midge host-seeking activity in winter may be underestimated as light traps will not capture midges active before sunset. This is particularly important given that peak activity often occurred before or during sunset from October through February, including before sunset on nearly 25% of nights in December and January.

### Supplementary Information

**Additional file 1: Fig. S1. **Host-seeking activity (2018–2019). **Fig. S2.** Host-seeking activity (2019–2020). **Fig. S3.** Host-seeking activity (2020–2021). **Table S1. **Summary of possible values for each independent variable.

**Additional file 2: Table S2.** Host-seeking activity of *C. sonorensis* and weather information.

## Data Availability

The datasets used and/or analyzed during the current study are available in Additional file 2.
