# Supplementary material for: Seasonal change and influence of environmental variables on host-seeking activity of the biting midge Culicoides sonorensis at a southern California dairy, USA
Source: Parasit Vectors. 2024 May 10;17:212. doi: 10.1186/s13071-024-06290-w (PMC11083819; doi:10.1186/s13071-024-06290-w)
Supplement: Supplementary file 1 — Additional file 1: Fig. S1. Host-seeking activity (2018–2019). Fig. S2. Host-seeking activity (2019–2020). Fig. S3. Host-seeking activity (2020–2021). Table S1. Summary of possible values for each independent variable. [file 13071_2024_6290_MOESM1_ESM.pdf]

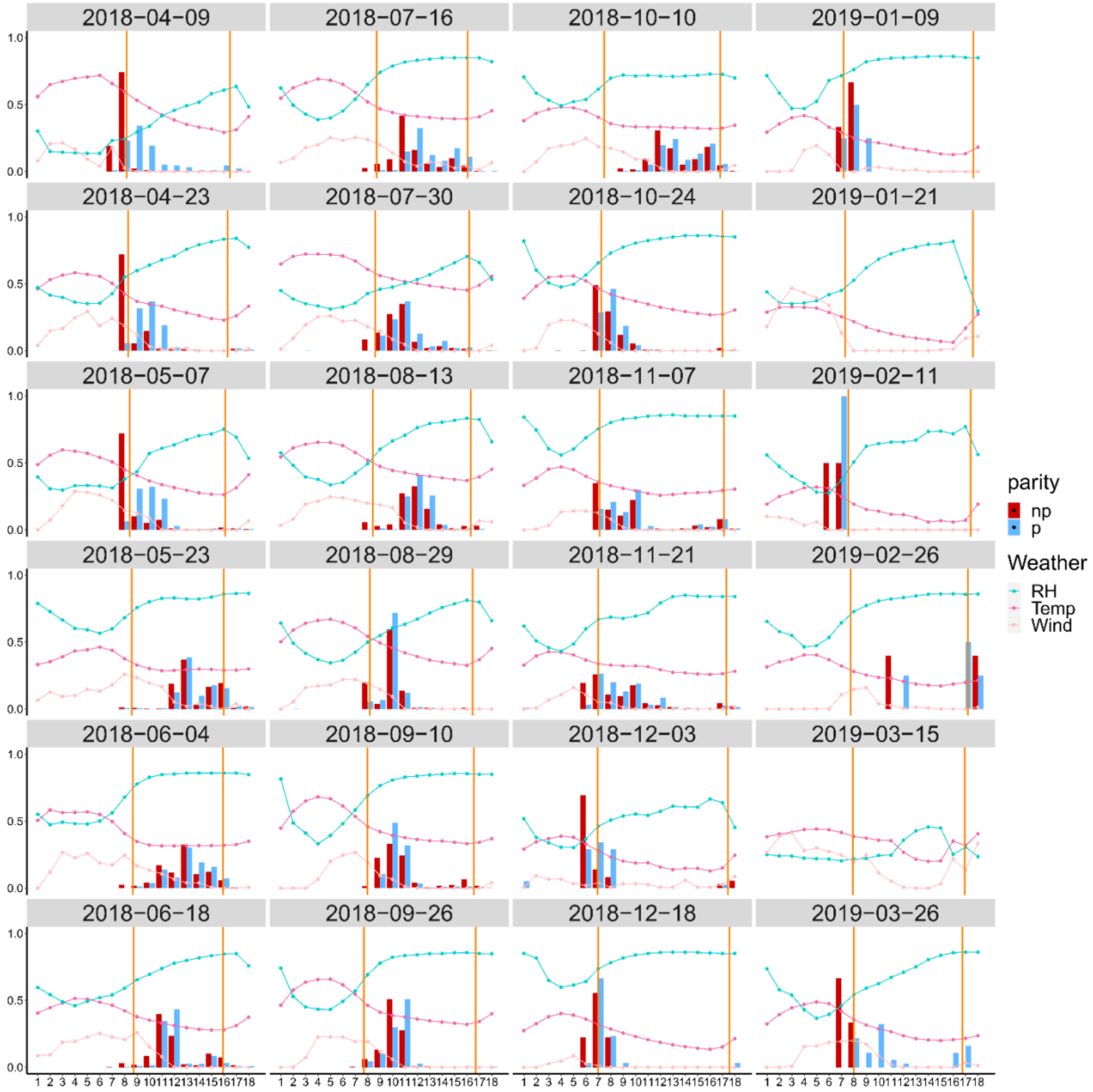

Fig. S1. Host-seeking activity (2018-2019). Columns show host-seeking parous (blue) and nulliparous (red) captured during each 80-min trapping interval (1-18) as a proportion of the total number of midges captured over the 24-h collection period starting at 8:45AM on the date indicated above each figure. Environmental factors including relative humidity (RH; blue line), temperature (Temp; dark pink line), and wind speed (Wind; light pink line) are provided as unitless features for general comparison to host-seeking activity patterns on each collection date. Two orange vertical lines represent sunset and sunrise.

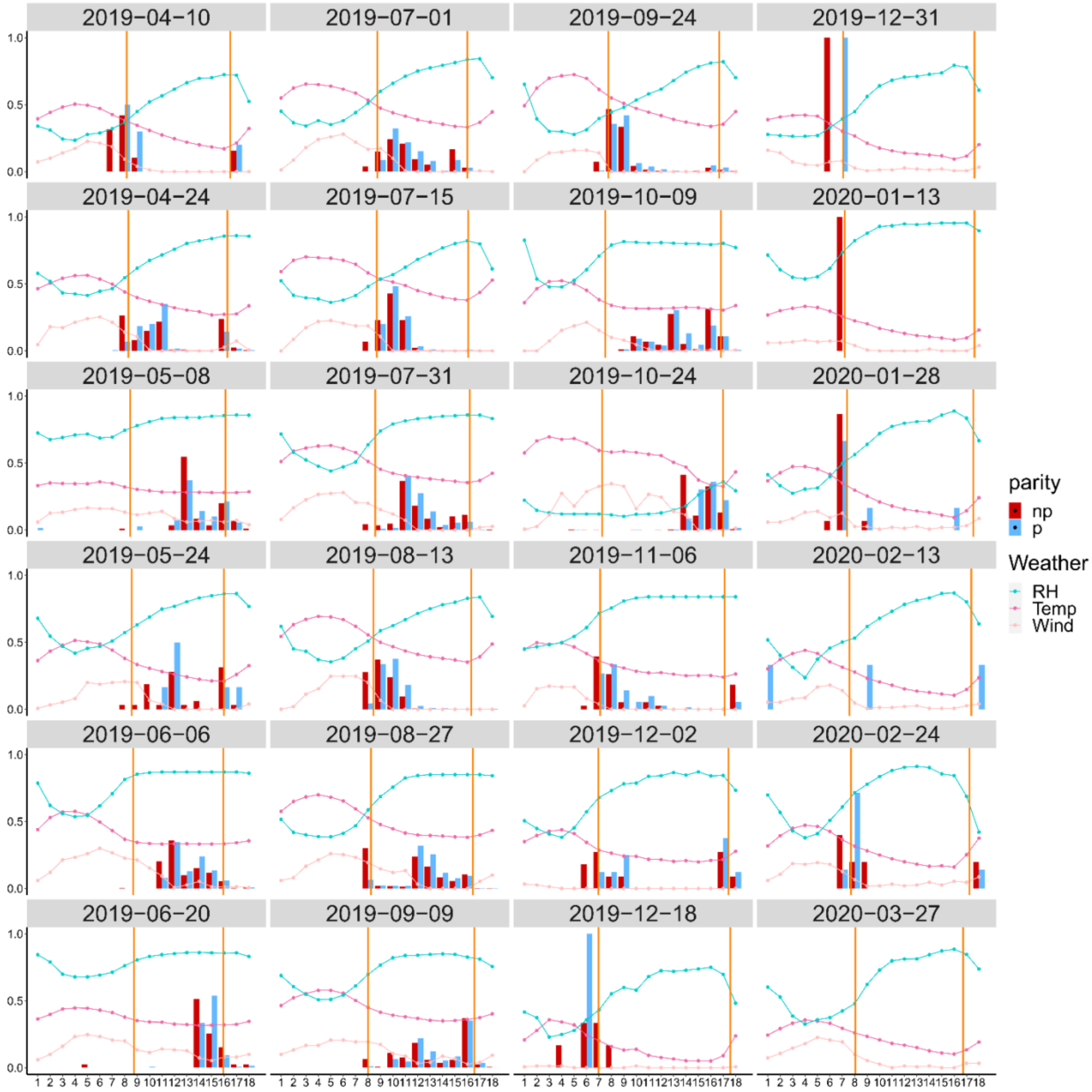

Fig. S2. Host-seeking activity (2019-2020). Columns show host-seeking parous (blue) and nulliparous (red) captured during each 80-min trapping interval (1-18) as a proportion of the total number of midges captured over the 24-h collection period starting at 8:45AM on the date indicated above each figure. Environmental factors including relative humidity (RH; blue line), temperature (Temp; dark pink line), and wind speed (Wind; light pink line) are provided as unitless features for general comparison to host-seeking activity patterns on each collection date. Two orange vertical lines represent sunset and sunrise.

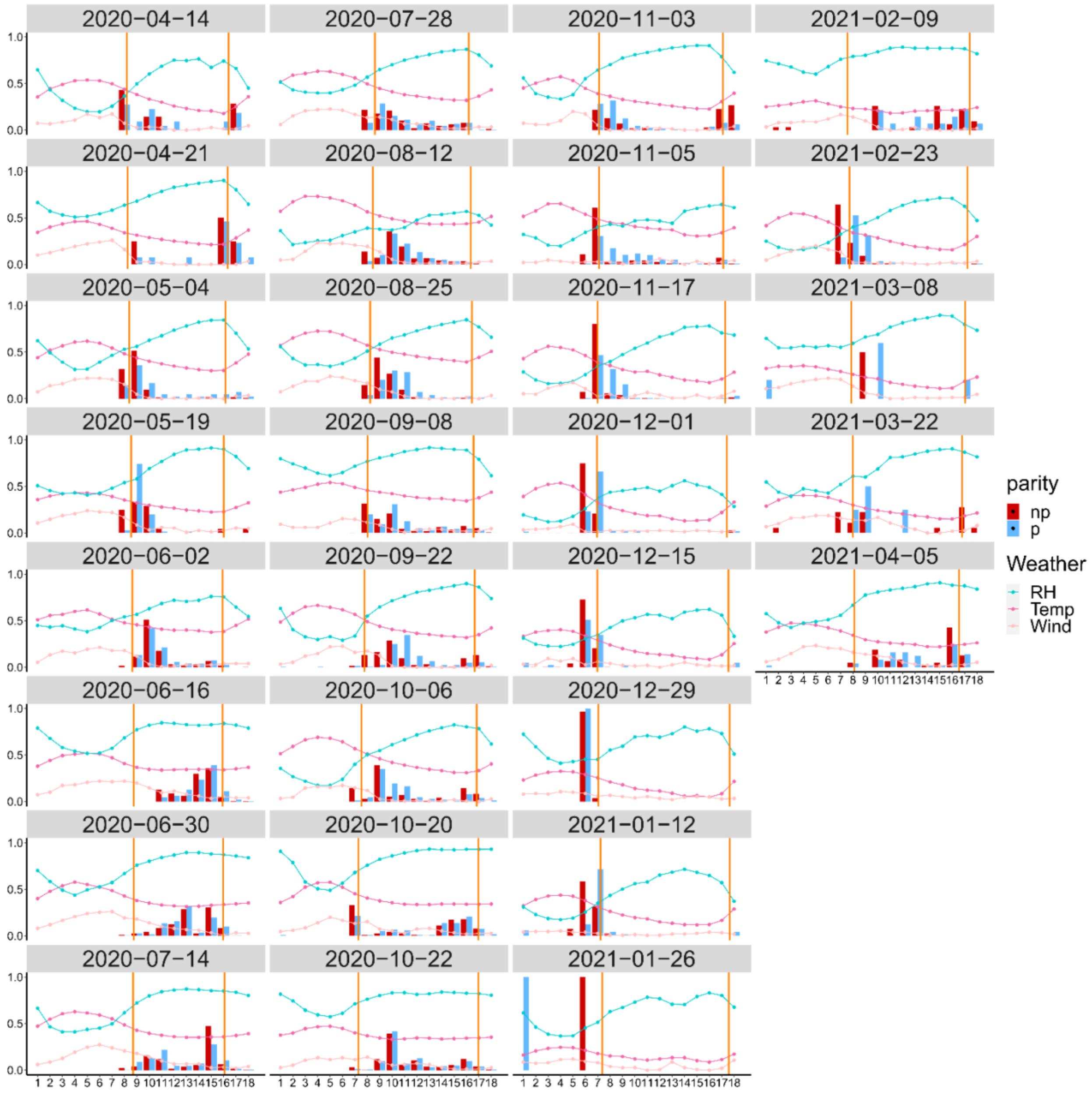

Fig. S3. Host-seeking activity (2020-2021). Columns show host-seeking parous (blue) and nulliparous (red) captured during each 80-min trapping interval (1-18) as a proportion of the total number of midges captured over the 24-h collection period starting at 8:45AM on the date indicated above each figure. Environmental factors including relative humidity (RH; blue line), temperature (Temp; dark pink line), and wind speed (Wind; light pink line) are provided as unitless features for general comparison to host-seeking activity patterns on each collection date. Two orange vertical lines represent sunset and sunrise.

Table S1. Summary of observed values for each independent variable.

	Min.	1st Qu.	Median	Mean	3rd Qu.	Max.
T_{h-1}	10.7	20.2	27.8	26.4	32.3	38.7
T_{l-1}	0.1	8.5	12.8	12.1	16.0	23.1
R_{h-1}	57.0	82.0	86.0	84.0	88.0	97.0
R_{l-1}	10.0	26.5	38.0	39.6	50.5	84.0
T_h	12.7	22.6	27.7	27.1	32.0	37.2
T_l	2.0	8.3	13.4	12.2	16.2	22.4
R_h	38.0	83.0	86.0	83.2	87.8	97.0
R_l	10.0	27.3	37.0	36.2	44.8	66.0
W_h	0.5	3.3	4.2	3.9	4.5	7.6
S_h	330.5	721.0	917.0	866.4	997.8	1253.0
T_s	8.9	17.3	20.2	20.9	24.7	30.7
R_s	12.0	40.8	55.1	54.3	65.5	81.4
W_s	0.0	1.3	2.3	2.1	2.9	4.9
M_n	0.0	4.0	7.0	7.1	10.0	14.0

T_{h-1}, T_{l-1}: the high (maximum) and low (minimum) temperature during the 24-h prior to start of the collection period.

T_h, T_l: the high (maximum) and low (minimum) temperature during the collection period.

R_{h-1}, R_{l-1}: high and low relative humidity during the 24-h prior to start of the collection period.

R_h, R_l: high and low relative humidity during the collection period.

S_h: high solar intensity during the collection period.

W_h: high wind speed during the collection period.

T_s, R_s, W_s: the temperature, relative humidity, and wind speed at sunset on each trapping date.

M_n: moon phase represented by the number of days to the closest new moon.
